# Rapid improvement of hepatic steatosis and liver stiffness after metabolic/bariatric surgery: a prospective study

**DOI:** 10.1038/s41598-024-67415-w

**Published:** 2024-07-30

**Authors:** Larissa Nixdorf, Lukas Hartl, Stefanie Ströhl, Daniel Moritz Felsenreich, Magdalena Mairinger, Julia Jedamzik, Paula Richwien, Behrang Mozayani, Georg Semmler, Lorenz Balcar, Michael Schwarz, Mathias Jachs, Nina Dominik, Christoph Bichler, Michael Trauner, Mattias Mandorfer, Thomas Reiberger, Felix B. Langer, David Josef Maria Bauer, Gerhard Prager

**Affiliations:** 1 Division of Visceral Surgery, Department of General Surgery, Medical University of Vienna, Vienna, Austria; 2 Division of Gastroenterology and Hepatology, Department of Medicine III, Medical University of Vienna, Vienna, Austria; 3 Department of Pathology, Medical University of Vienna, Vienna, Austria

**Keywords:** Bariatric surgery, Metabolic surgery, MASLD, MASH, Fibroscan, Liver stiffness, Controlled attenuation parameter, Liver fibrosis, Non-alcoholic fatty liver disease, Non-alcoholic steatohepatitis, Obesity

## Abstract

Metabolic dysfunction-associated steatotic liver disease (MASLD) and related steatohepatitis (MASH) are common among obese patients and may improve after metabolic/bariatric surgery (MBS). 93 Patients undergoing MBS in 2021–2022 were prospectively enrolled. Liver stiffness measurement (LSM; via vibration-controlled transient elastography [VCTE], point [pSWE] and 2D [2DSWE] shear wave elastography) and non-invasive steatosis assessment (via controlled attenuation parameter [CAP]) were performed before (baseline [BL]) and three months (M3) after surgery. 93 patients (median age 40.9 years, 68.8% female, median BL-BMI: 46.0 kg/m^2^) were included. BL-liver biopsy showed MASLD in 82.8% and MASH in 34.4% of patients. At M3 the median relative total weight loss (%TWL) was 20.1% and the median BMI was 36.1 kg/m^2^. LSM assessed by VCTE and 2DSWE, as well as median CAP all decreased significantly from BL to M3 both in the overall cohort and among patients with MASH. There was a decrease from BL to M3 in median levels of ALT (34.0 U/L to 31 U/L; p = 0.025), gamma glutamyl transferase (BL: 30.0 to 21.0 U/L; p < 0.001) and MASLD fibrosis score (BL: − 0.97 to − 1.74; p < 0.001). Decreasing LSM and CAP, as well as liver injury markers suggest an improvement of MASLD/MASH as early as 3 months after MBS.

## Introduction

Metabolic dysfunction-associated steatotic liver disease (MASLD), which may progress to metabolic dysfunction-associated steatohepatitis (MASH), has become a global healthcare burden and its prevalence is constantly increasing^1,2^. MASLD is strongly associated with obesity, diabetes and hyperlipidemia and is hence considered the hepatic manifestation of the metabolic syndrome^3^. To this day there are no pharmacological treatments specifically approved for MASLD/MASH^4^. However, in obese patients, weight loss substantially improves liver health by reducing insulin resistance, decreasing hepatic steatosis, and improving liver enzymes^5,6^. Lifestyle interventions that promote weight loss, such as physical activity and dietary changes are thus, effective in improving liver health in individuals with MASLD/MASH^7,8^. Unfortunately, long-term weight loss is often not sufficiently achieved by lifestyle interventions^9^.

Metabolic/Bariatric surgery (MBS) is now increasingly recognized as effective treatment for MASLD/MASH^10^, with particularly strong evidence for its effectiveness generated from studies involving patients with obesity^11,12^. Recently, a multicenter randomized trial was conducted, demonstrating the superiority of MBS to lifestyle intervention plus best medical care in the therapy of obese patients with MASH^13^. The underlying mechanisms include weight loss, metabolic improvements, beneficial effects on the gut microbiota and their metabolic function, gut hormones and systemic inflammation, and amelioration of adipokine levels^12–14^. However, we have also observed new-onset of hepatic dysfunction after MBS in some patients^15^. Thus, additional data on the course of MASLD/MASH after MBS is needed.

While liver biopsy still represents the gold-standard for liver disease evaluation, it is an invasive procedure associated with morbidity and even mortality^16^. Consequently, non-invasive liver stiffness measurement (LSM) is now widely used as a surrogate marker for liver fibrosis^16^. LSM can be performed via vibration-controlled transient elastography (VCTE), point-shear wave elastography (pSWE) or 2D shear wave elastography (2DSWE)^16^. While VCTE is well-validated and widely available^17,18^, pSWE and 2DSWE can be integrated in ultrasound devices and therefore are promising technologies combining LSM with liver imaging^16^. The dedicated VCTE device (FibroScan®, Echosens, France) allows for simultaneous assessment of liver steatosis by controlled attenuation parameter (CAP)^19^.

The aim of this prospective, observational study was to investigate the early trajectories of (i) LSM as assessed by VCTE, pSWE and 2DSWE, (ii) CAP and (iii) lab-based surrogates of MASLD/MASH severity before and 3 months after MBS.

## Material and methods

### Study design

Patients who underwent MBS at the Vienna General Hospital between January 2021 and December 2022 were prospectively enrolled and characterized. Examinations were performed both preoperatively (BL) and during the first postoperative follow-up visit, scheduled at 3 months (M3). Patients lost to follow-up prior to M3 after MBS were excluded from further analysis. Weight loss outcomes were quantified in terms of excess weight loss percentage (EWL%), utilizing a reference standard of 25 kg/m^2^ BMI and percentage of total weight loss (%TWL) relative to initial preoperative body weight.

The study included patients who underwent one of the following metabolic/bariatric procedures: Single Anastomosis Duodeno-Ileal Bypass with Sleeve Gastrectomy (SADI-S), One Anastomosis Gastric Bypass (OAGB), Roux-en-Y Gastric Bypass (RYGB) or Sleeve Gastrectomy (SG). The surgical method was decided based on BMI, comorbidities, the occurrence of reflux, patient preference and other factors.

### Surgical method

All surgical procedures followed standardized protocols and techniques were previously described^20–23^. Briefly, each surgery involved the use of 5 trocars, except in cases of SADI-S where a 6th trocar above the symphysis was occasionally necessary.

For SG, a sleeve was created using a 12 mm bougie starting approximately 2–4 cm orally from the pylorus, using 2 black cartridges at the beginning for the thicker tissue near the pylorus, followed by purple and beige cartridges. After resection, the gastrosplenic and gastrocolic ligament were refixed at the staple line.

For SADI-S, a 16 mm bougie was used to create the pouch. After this, 300 cm of small bowel were measured from the ileocecal transition for the common limb, followed by creation of a hand-sewn duodenoileostomy.

In OAGB and RYGB, the gastric pouch was formed long and narrow using the same bougie size (12 mm) as in SG. The pouch in OAGB was slightly longer than that in RYGB. The biliopancreatic limb was measured 150 cm long in both procedures. For RYGB, the jejunojejunostomy was set at 70 cm of the alimentary limb. The Peterson space was closed in both procedures, additionally the mesenteric gap was closed in RYGB.

### Surgical complications

A detailed description of the surgical procedures is provided in the Supplementary material. Postoperative complications were classified according to the Clavien Dindo Classification (CDC)^24^.

### Liver stiffness measurement and liver steatosis assessment

Patients underwent VCTE (FibroScan®, Echosens, France) for LSM and CAP evaluations, as well as point shear-wave elastography (pSWE) and 2D shear wave elastography (2DSWE) (Siemens Medical Solutions USA, Inc., Issaquah, WA) using the 5C1 probe on the Siemens ACUSON Sequoia system (Software Revision VA30) using a standardized protocol. Prior to examinations, patients fasted for at least 4 h.

Adhering to the 2020 SRU Guidelines^25^ and local expertise^26,27^ measurements were performed in a right intercostal space, with patients in a supine position and breath held at mid-respiration. For pSWE and 2DSWE, the probe was positioned 1.5 cm beneath the liver capsule, as directed by the ultrasound machine's built-in guide, while avoiding larger structures or vessels in the region of interest. For VCTE the medium (M) or extra-large (XL) probe was chosen according to the built-in probe selection tool. Reliable VCTE measurements were defined as LSM < 7.1 kPa or interquartile range/median (IQR/med) ≤ 0.3 for LSM ≥ 7.1 kPa and as IQR/med ≤ 0.3 dB/m for CAP corresponding to previously established criteria^18,19^.

### Non-invasive liver fibrosis and liver steatosis stages

For grading of liver fibrosis stage as assessed by VCTE, the following MASLD-specific cut-offs^16,17,28^ were used: F0/F1: < 8.0 kPa, F2: 8.0–9.9 kPa, F3/F4: ≥ 10.0 kPa. Liver steatosis was staged by CAP according to 3 different proposed CAP cutoffs: firstly following Karlas et al.^29^: S0: < 248 dB/m, S1: 248–267 dB/m, S2: 268–279 dB/m and S3: ≥ 280 dB/m; secondly according to the cutoffs proposed by Runge et al.^30^: S0: < 260 dB/m, S1: 260–295 dB/m, S2: 296–333 dB/m and S3: ≥ 334 dB/m; thirdly using the cutoffs proposed by Naveau et al.^31^: S0: < 308 dB/m, S1: 308–334 dB/m, S2: 335–340 dB/m and S3: ≥ 341 dB/m.

### Liver biopsy

A protocol liver biopsy was conducted during bariatric surgery. The biopsies were analyzed in a standardized manner at the Department of Pathology at the Medical University of Vienna following previously established methods^32^. MASLD was defined as hepatic steatosis in the presence of at least one metabolic risk factor^2^ and MASH was diagnosed according to the algorithm proposed by Bedossa et al.^33^. Moreover, the non-alcoholic fatty liver disease activity score (NAS) was assessed.

### Laboratory parameters

Laboratory parameters were analyzed at the ISO-certified Department of Laboratory Medicine at the Vienna General Hospital. Standard laboratory methods were used for the measurements of routine laboratory parameters that were assessed before (BL) and M3 after MBS.

Standard laboratory thresholds for men and women were used as upper limit of normal (ULN) for parameters of hepatocellular (aspartate aminotransferase [AST], alanine aminotransferase [ALT]) and cholestatic liver injury (gamma glutamyl transferase [GGT], bilirubin). Hepatocellular liver injury was defined as increase of AST or ALT > 3xULN, while cholestatic liver injury was defined as increase of GGT or bilirubin > 2xULN as modified from the definition of the American College of Gastroenterology Clinical Guideline^34,35^.

### Statistical analysis

Continuous data are presented as median and interquartile range (IQR), while categorical variables are reported as the number (n) and proportion (%) of patients exhibiting the parameter of interest. Wilcoxon's signed-rank test was employed for comparing metric variables in dependent samples, and Pearson's Chi-squared test was used for comparing categorical variables across multiple groups. Statistical analyses were performed using IBM SPSS 27.0 (IBM, Armonk, NY, USA) and GraphPad Prism 8 (GraphPad Software, La Jolla, CA, USA). A two-sided p-value of < 0.05 was deemed statistically significant.

### Ethics

This study received approval from the Medical University of Vienna's ethics committee (No. 1509/2020) and adhered to the current version of the Helsinki Declaration and the institution's Guidelines on Good Scientific Practice. All participating patients provided written informed consent prior to study inclusion.

## Results

### Patient characteristics

**Figure 1:**
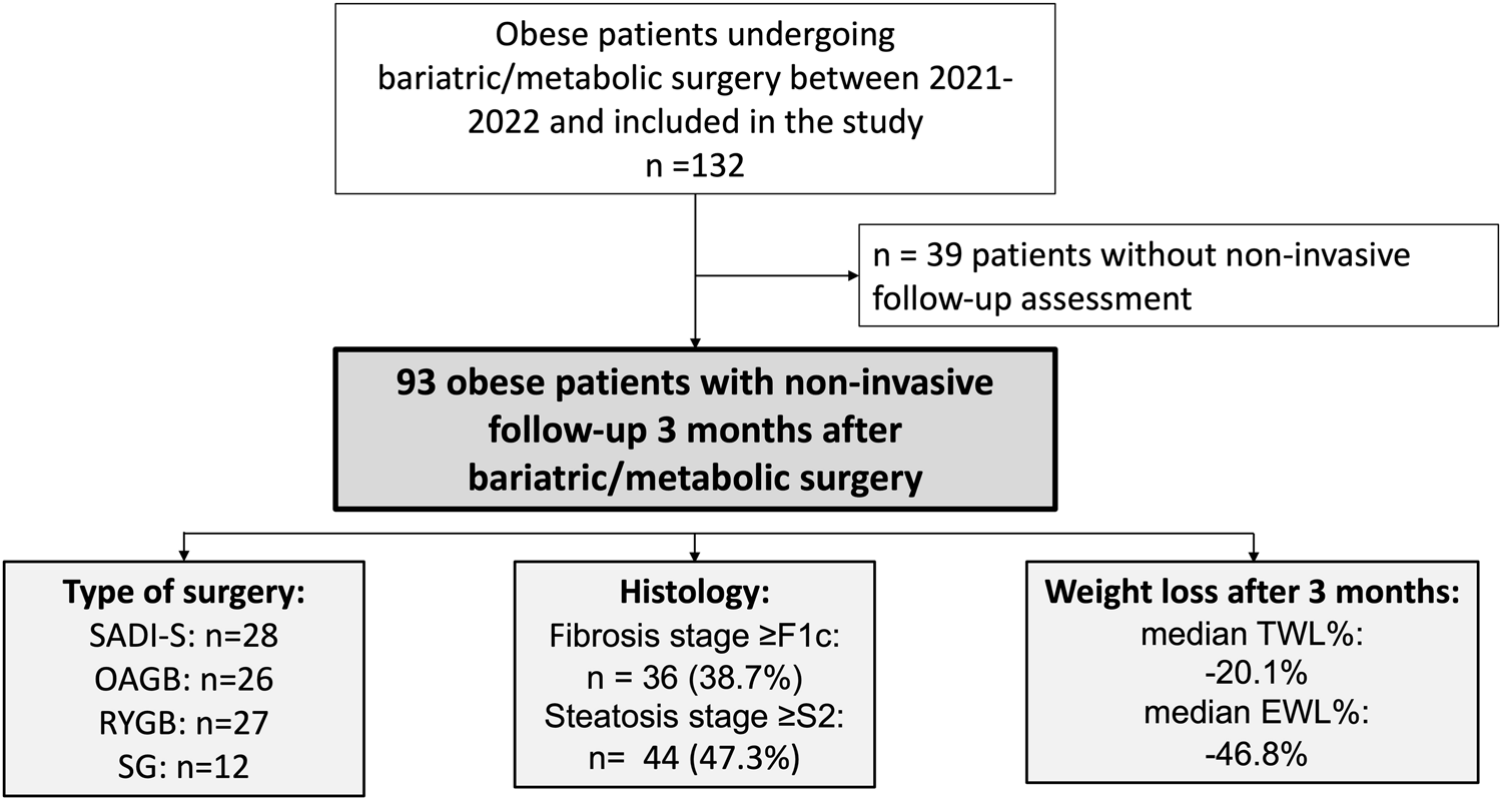
Patient flowchart. *%EWL* relative excess weight loss, *SADI-S* single anastomosis duodenoileal bypass with sleeve gastrectomy, *OAGB* one anastomosis gastric bypass, *RYGB* roux-en-Y gastric bypass, *SG* sleeve gastrectomy, *%TWL* relative total weight loss, *VCTE* vibration-controlled transient elastography.

The cohort building process is depicted in Fig. 1. Overall, 93 patients (SADI-S: n = 28; OAGB: n = 26; RYGB: n = 27; SG: n = 12) with a median age of 40.9 years and female predominance (68.8%) were included in the final cohort. Detailed patient characteristics are provided in Table-1. The median BMI was 46.0 kg/m^2^.

### Liver histology

In liver biopsy, 44.1% (n = 41) of patients had histological fibrosis stage ≥ F1c and 47.3% (n = 44) of patients had histological stage ≥ S2. 82.8% (n = 77) patients were diagnosed with MASLD, while 34.4% (n = 32) had MASH. The median NAS was 3.0. Inflammation was present in 49.4% (n = 46) of patients and ballooning in 53.8% (n = 50) of patients.

### LSM and CAP at BL

Assessed via VCTE-LSM, 26.4% of patients had ≥ F2 (in patients with reliable VCTE-LSM at baseline [BL]; n = 19/72). Median BL-LSM increased numerically with liver disease severity (no liver disease: 4.8 [IQR 4.1–8.3] kPa vs. MASLD: 5.7 [IQR 4.6–7.7] kPa vs. MASH: 6.7 [4.9–9.8] kPa; p = 0.449), while median BL-CAP increased significantly (no liver disease: 264.5 [IQR 222.1–318.5] kPa vs. MASLD: 329.0 [IQR 290.0–377.0] kPa vs. MASH: 340.5 [302.5–384.5] kPa; p = 0.002).

### Liver steatosis stages at BL assessed by histology and CAP

Among patients with reliable CAP at BL, 15.0% (n = 12) of patients had steatosis stage S0 in liver histology, while 33.7% (n = 27) had S1, 28.8% (n = 23) had S2 and 22.5% (n = 18) had S3. In comparison, Table S1 lists the prevalence of non-invasively-assessed liver steatosis stages staged by different previously proposed CAP cut-offs that have been previously proposed for patients with MASLD. The CAP steatosis stage cutoffs proposed by Karlas et al.^29^ had the highest correlation with liver steatosis in histology (ρ = 0.312; p = 0.005), but classified 82.5% (n = 66) of patients with reliable CAP at BL as steatosis stage ≥ S2, which was markedly higher than the proportion of patients with ≥ S2 in liver histology (51.7%). In contrast, the cutoffs proposed by Naveau et al.^31^ classified 48.8% of patients as steatosis stage ≥ S2 (i.e., comparable to histological assessment), however, also misclassified a considerable number of patients as steatosis stage S0 (S0: n = 29 [36.3%] compared to n = 12 [15.0%] in liver histology), yielding an overall correlation of only ρ = 0.221 (p = 0.049) with liver steatosis.

### Surgical outcomes and complications

After M3 median TWL% was 20.1% and median EWL% was 46.8% (Table 1). The overall complication rate was 15.1%; 53.3% of complications were early complications (within 30 days) and 46.7% were late complications (> 30 days after MBS). Of the eight early postoperative complications, 75% were classified as CDC grade IIIb and 25% as grade I. Out of the patients who required revision surgery within 30 days, three being attributed to bleeding, two to dysphagia, and one to leakage at the gastrojejunostomy site. Additionally, seven cases of late complications were observed, occurring after the first postoperative month. Of those, 57% classified as CDC Grade II, requiring intravenous substitutional therapy due to iron deficiency and 14% as CDC grade I, necessitating elongated treatment with proton pump inhibitors due to an anastomotic ulcer at 9 moths. 28% were classified as CDC grade IIIb requiring revision surgery. One needed endoscopic esophageal stent treatment for two weeks due to dysphagia after SG, while the other one was converted to RYGB due to biliary reflux after SADI-S.

**Table 1:** Patient characteristics and weight change after metabolic/bariatric surgery.

Patient characteristics	All patients (n = 93)
Sex, female/male (% female)	64/29 (68.8%)
Age, years (IQR)	40.9 (32.5–52.1)
BMI at BL, kg/m^2^ (IQR)	46.0 (41.6–51.3)
BMI after 3 M, kg/m^2^ (IQR)	36.1 (31.5–40.9)
Relative weight change after 3 M, % (IQR)	20.1 (16.5–24.9)
Excess weight loss after 3 M, % (IQR)	46.8 (35.9–60.6)
Complications, n (%)	
Early complications, n (%)	8 (8.6%)
Late complications, n (%)	7 (7.3%)
Histology	
Fibrosis stage ≥ F1c, n (%)	41 (44.1%)
Steatosis stage ≥ S2, n (%)	44 (47.3%)
MASLD, n (%)	77 (82.8%)
MASH, n (%)	32 (34.4%)
NA-score, median (IQR)	3.0 (1.0–4.0)
Inflammation, n (%)	46 (49.4%)
Ballooning, n (%)	50 (53.8%)
Reliable LSM/VCTE at BL, n (%)	72 (77.4%)
LSM/VCTE at BL, kPa (IQR)	5.8 (4.6–8.2)
LSM/VCTE stage ≥ F2 at BL, n (%)	19 (26.4%)
Reliable CAP at BL, n (%)	80 (86.0%)
CAP at BL, dB/m (IQR)	328.0 (290.5–378.0)
AST at BL, U × L^−1^ (IQR)	23.0 (19.0–31.0)
ALT at BL, U × L^−1^ (IQR)	34.5 (25.0–51.0)
Bilirubin at BL, mg × dL^−1^ (IQR)	0.5 (0.4–0.6)
Platelets at BL, G × L^−1^ (IQR)	255.5 (220.5–311.0)
Comorbidities at BL	
Type 2 diabetes, n (%)	25 (26.9%)
Arterial hypertension, n (%)	41 (44.1%)
Dyslipidemia, n (%)	23 (24.7%)
COPD, n (%)	5 (5.4%)
Sleep apnea, n (%)	14 (15.1%)

*3M* 3 months, *BL* baseline, *BMI* body mass index, *CAP* controlled attenuation parameter, *COPD* chronic obstructive pulmonary disease, *LSM* liver stiffness measurement, *MASH* metabolic dysfunction-associated steatohepatitis, *MASLD* metabolic dysfunction-associated steatotic liver disease.

### Success rate and reliability of VCTE and CAP

In total, longitudinal LSM was available in 56 patients. Among these patients, baseline or follow-up VCTE was unsuccessful or unreliable in 23.2% (n = 13), which is comparable to the success rate of 77.4% in the entire cohort. Baseline or follow-up CAP was unsuccessful or unreliable in 16.1% (n = 9) patients. Again, this corresponded well with the success rate of 82.5% in the entire cohort. In comparison, pSWE and 2DSWE, which were longitudinally performed in 54 patients, were successful in 94.4% (n = 51) of patients.

### Changes of LSM and CAP after MBS

**Figure 2:**
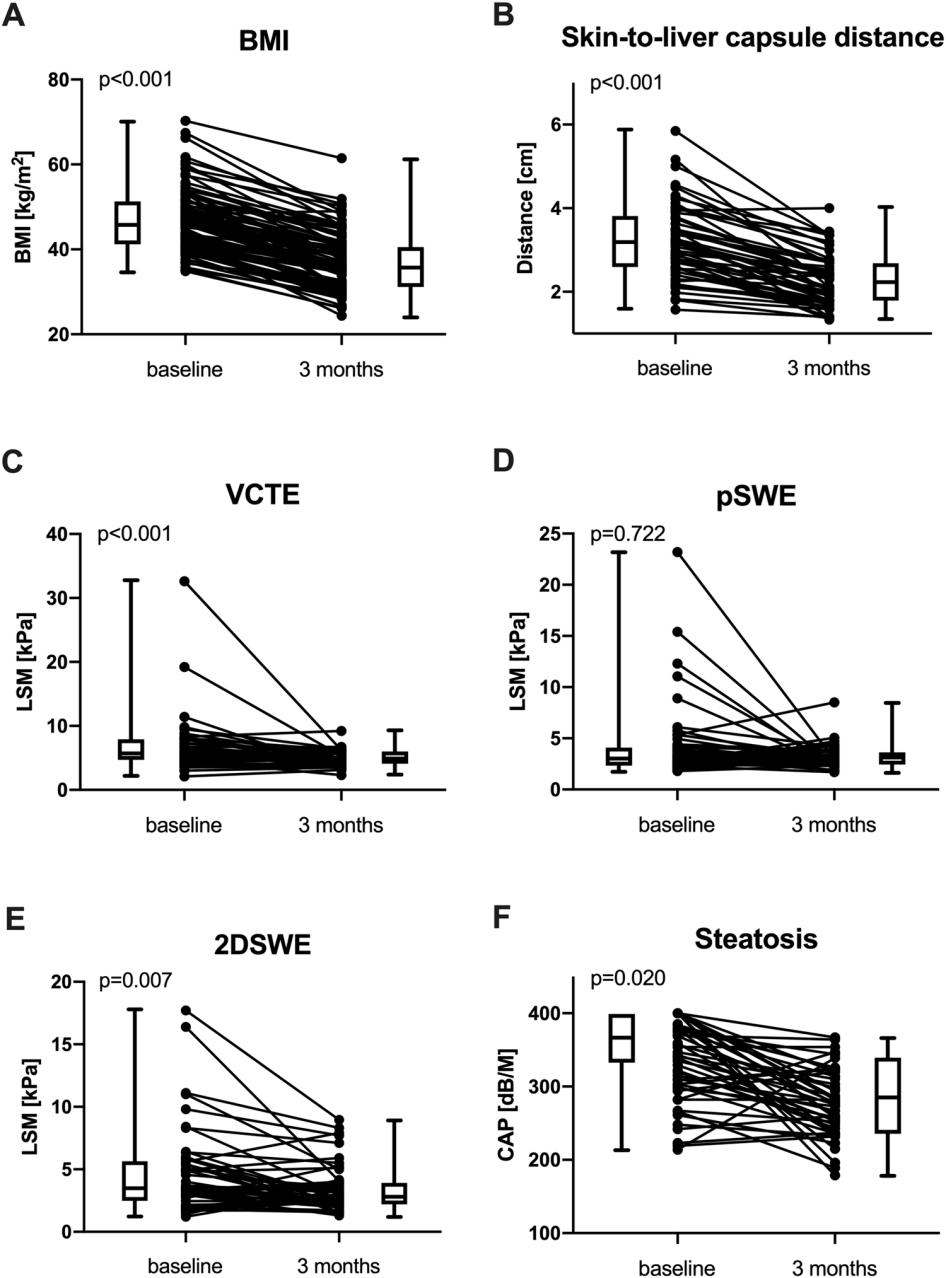
Comparison of (**A**) BMI, (**B**) skin-to-liver capsule distance, liver stiffness measured by (**C**) VCTE, (**D**) pSWE and (**E**) 2D SWE, as well as (**F**) CAP at baseline and after 3 months. *2DSWE* 2D share wave elastography, *BMI* body mass index, *CAP* controlled attenuation parameter, *LSM* liver stiffness measurement, *pSWE* point share wave elastography.

Importantly, a significant decrease in LSM was observed by VCTE (BL: 5.6 kPa vs. M3: 4.8 kPa; p < 0.001), and 2DSWE (BL: 3.5 kPa vs. M3: 2.9 kPa; p = 0.018), while median LSM assessed by pSWE did not significantly change 3 months after MBS (BL: 3.1 kPa vs. M3: 3.2 kPa; p = 0.904) (Table 2, Fig. 2).

**Table 2:** Trajectory of LSM and CAP after metabolic/bariatric surgery.

All patients	At baseline	After 3 months	p-value
LSM by vibration-controlled transient elastography, kPa (IQR)^1^	5.6 (4.6–7.8)	4.8 (4.0–5.9)	** < 0.001**
LSM by point shear wave elastography, kPa (IQR)	3.1 (2.4–4.1)	3.2 (2.5–3.7)	0.904
LSM by 2D shear wave elastography, kPa (IQR)	3.5 (2.5–5.6)	2.9 (2.3–4.0)	**0.018**
Controlled attenuation parameter, dB × m^−1^ (IQR)^2^	341.0 (297.0–384.0)	277.0 (243.0–320.0)	** < 0.001**

*CAP* controlled attenuation parameter, *LSM* liver stiffness measurement.

^1^Indicated as median of reliable longitudinal VCTE measurements. Available in n = 43 patients.

^2^Indicated as median of reliable longitudinal CAP measurements. Available in n = 47 patients.

There was a significant decrease of fibrosis stage as assessed by VCTE (p = 0.007; Table 3). While 20% (n = 9/43) of patients with longitudinal reliable LSM/VCTE had ≥ F2 (i.e. LSM ≥ 8.0 kPa) at baseline, only one patient (2.3%) with ≥ F2 remained after 3 months.

**Table 3:** Short-term changes of VCTE-based fibrosis risk and CAP-based steatosis assessment after bariatric/metabolic surgery.

All patients	At baseline	After 3 months	p-value
VCTE-LSM fibrosis risk^1^			0.007
F0/F1 < 8 kPa, n (%)	34 (79.0%)	42 (97.7%)	
F2 8–9.9 kPa, n (%)	6 (14.0%)	1 (2.3%)	
F3/F4 ≥ 10 kPa, n (%)	3 (7.0%)	0 (0.0%)	
CAP steatosis stage defined by Karlas et al.^29^ ^2^			< 0.001
S0 (< 248 dB/m), n (%)	4 (8.5%)	12 (25.5%)	
S1 (248–267 dB/m), n (%)	4 (8.5%)	9 (19.2%)	
S2 (268–279 dB/m), n (%)	0 (0.0%)	5 (10.6%)	
S3 (≥ 280 dB/m), n (%)	39 (83.0%)	21 (44.7%)	
CAP steatosis stage defined by Runge et al. ^30^ ^2^			< 0.001
S0 (< 260 dB/m), n (%)	5 (10.6%)	18 (38.3%)	
S1 (260–295 dB/m), n (%)	6 (12.8%)	13 (27.7%)	
S2 (296–333 dB/m), n (%)	10 (21.3%)	10 (21.3%)	
S3 (≥ 334 dB/m), n (%)	26 (55.3%)	6 (21.7%)	
CAP steatosis stage defined by Naveau et al. ^31^ ^2^			< 0.001
S0 (< 308 dB/m), n (%)	15 (31.9%)	33 (70.2%)	
S1 (308–334 dB/m), n (%)	6 (12.8%)	8 (17.0%)	
S2 (335–340 dB/m), n (%)	2 (4.3%)	1 (2.1%)	
S3 (≥ 341 dB/m), n (%)	24 (51.0%)	5 (10.6%)	

*CAP* controlled attenuation parameter, *LSM* liver stiffness measurement, *VCTE* vibration-controlled transient elastography.

^1^Indicated as fibrosis stage of reliable longitudinal VCTE measurements. Available in n = 43 patients.

^2^Indicated as median of reliable longitudinal CAP measurements. Available in n = 47 patients.

Furthermore, CAP significantly decreased after MBS (BL: 341.0 dB/m vs. M3: 277.0 dB/m; p < 0.001). Among patients with paired reliable longitudinal CAP measurement, steatosis stage as defined by Karlas et al.^29^ regressed significantly after 3 months (p < 0.001) with 83.0% exhibiting steatosis stage S2 (i.e. CAP ≥ 268 dB/m) at baseline, as compared to 55.3% 3 months after surgery. Importantly, as detailed in Table-3, similar results were achieved when applying the CAP cutoffs for steatosis stages proposed by Runge et al.^30^ and by Naveau et al.^31^.

### Trajectory of LSM and CAP in patients with MASH

Similar results were obtained in patients with MASH and longitudinal non-invasive assessment (Table S2, Fig. S1): There was a significant decrease in LSM at M3 assessed by VCTE (BL: 6.3 kPa vs. M3: 4.5 kPa; p = 0.015), and 2DSWE (BL: 3.4 kPa vs. M3: 2.4 kPa; p = 0.023). Moreover, median CAP markedly declined at M3 (BL: 347.5 dB/m vs. M3: 280.0 dB/m; p < 0.001).

### Trajectory of transaminases and metabolic parameters after MBS

**Figure 3:**
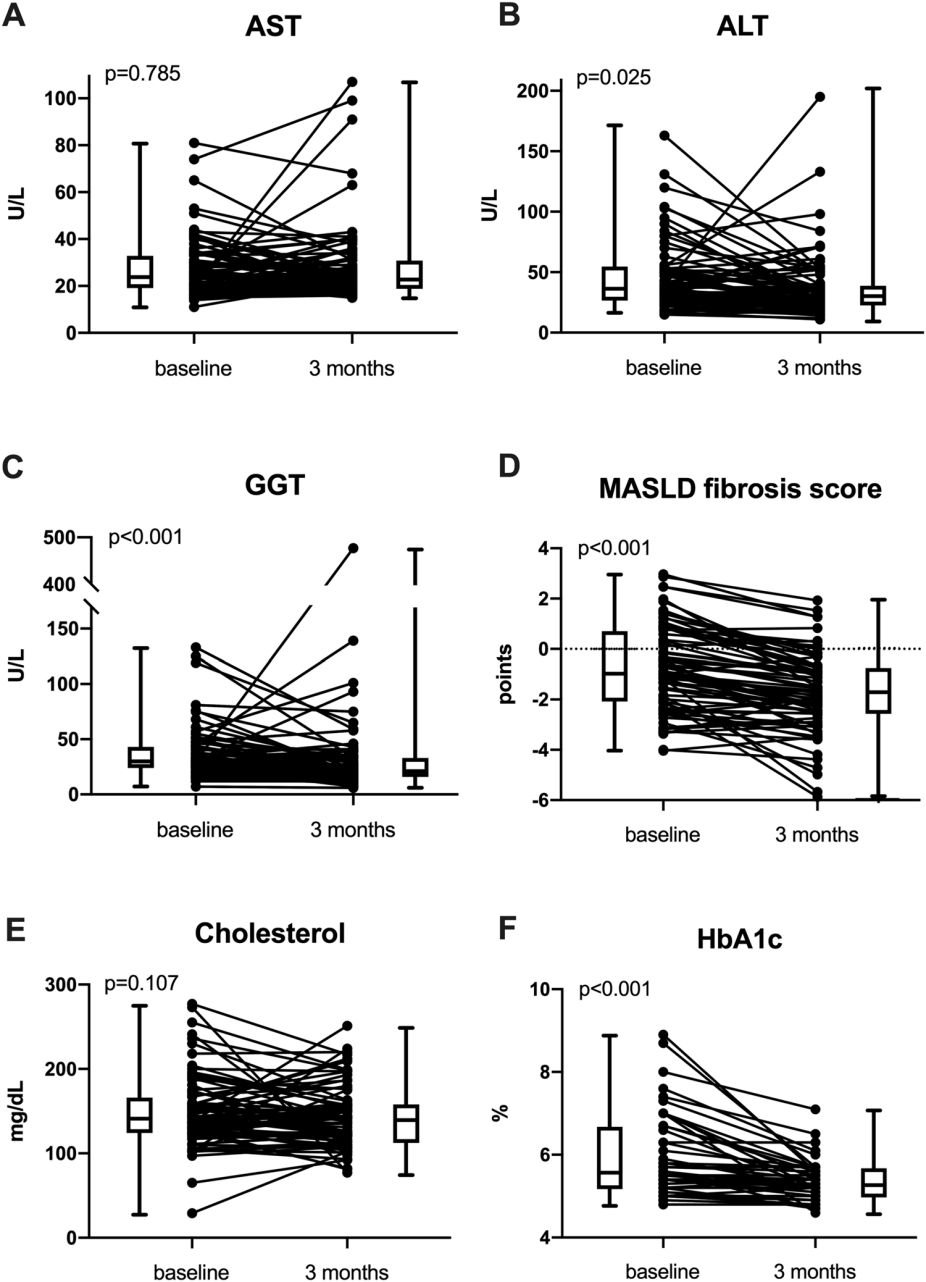
Comparison of (**A**) AST, (**B**) ALT, (**C**) GGT, (**D**) MASLD fibrosis score, (**E**) cholesterol and (**F**) HbA1c at baseline and after 3 months. *ALT* alanine aminotransferase, *AST* aspartate aminotransferase, *GGT* gamma glutamyl transferase, *HbA1c* glycated hemoglobin, *MASLD* metabolic dysfunction-associated liver disease.

Median levels of ALT significantly decreased 3 months after surgery (BL: 34.0 U/L vs. M3: 31.0 U/L; p = 0.025; Fig. 3, Table S2), while AST was not significantly different at BL and M3 after the bariatric procedure (BL: 24.0 U/L vs. M3: 23.0 U/L; p = 0.785). Moreover, median GGT decreased after MBS (BL: 30.0 U/L vs. M3: 21.0 U/L), while there was a slight increase in median bilirubin (BL: 0.5 U/L vs. M3: 0.6 U/L; p < 0.001).

There was a marked decrease in MASLD fibrosis score 3 months after surgery (BL: − 0.97 points vs. M3: − 1.74 points; p < 0.001). At the same time, AST to platelet ratio index (APRI) remained unchanged (p = 0.974). Comparable results were obtained in the subgroup of patients with MASH (Table S3, Fig. S1).

Median glycated hemoglobin (HbA1c) was significantly lowered 3 months after the bariatric procedure (BL: 5.6% vs. M3: 5.3%; p < 0.001) while median cholesterol levels did not significantly decline (BL: 143.0 mg/dL vs. M3: 142.0 mg/dL; p = 0.107).

While there were 1 patient with hepatocellular and 2 patients with cholestatic liver injury at baseline, at M3 after MBS laboratory signs of hepatocellular or cholestatic liver injury were found in 2 and 3 patients, respectively (Table S4). Notably, one patient with laboratory signs of hepatocellular liver injury 3 months after surgery also exhibited a bilirubin of 2.48 mg/dL (as compared to 0.49 mg/dL at BL). Importantly these findings were transient without deterioration of hepatic synthetic function or signs of liver failure and normalized without specific medical/therapeutic interventions.

## Discussion

Obesity and obesity-related comorbidities including MASLD/MASH^2^ represent significant challenges for health care on a global scale, with dramatically increasing prevalence^36^. While lifestyle modification and medical treatment for obesity and MASLD is challenging, MBS offers an effective option^10^. In this study, we investigate the short-term changes in LSM, CAP and laboratory-based surrogates of liver injury and metabolic parameters after MBS.

The overall composition of the cohort reflects a representative sample of patients undergoing MBS. After 3 months, the cohort exhibited an average excess weight loss (EWL) of 46.8%, with an average BMI reduction of 10 kg/m^2^ which is comparable to the existing literature concerning short term results after MBS^37^. Typically, the nadir weight is reached 12 to 18 months after MBS.

Around 25% of all patients suffered from type II diabetes preoperatively, with 32% of them requiring insulin therapy for treatment. After 3 months 28% of these patients showed an improvement regarding their medication. Also, the HbA1c levels of all patients decreased significantly. The development of MASLD is closely related to insulin resistance and the development of type II diabetes^38^. MBS and especially RYGB surgery showed an excellent performance in improvement or even remission of diabetes mellitus^39^.

The complication pattern observed in this study is common for a patient collective after MBS^40^. Compared to existing literature, the complication rate depicted here is relatively low^40,41^, but there were some severe complications requiring surgical correction.

Non-invasive tests (NIT) and particularly LSM has become increasingly relevant for the diagnosis of compensated advanced chronic liver disease (cACLD) and risk assessment for adverse clinical events such as hepatic decompensation or death^42^. In this study, we used VCTE as a well-established method for the non-invasive assessment of LSM^16,18,42^. Obesity is one of the most important factors associated with failure of VCTE-LSM^43^. Indeed, in our study in a cohort of severely obese individuals there was a high rate of failure or unreliable result of VCTE-LSM (i.e. 23.2%), which was comparable in patients undergoing different types of bariatric procedures.

Importantly, a significant decrease in LSM assessed by VCTE and 2DSWE was visible already 3 months after MBS. All but one patient with available longitudinal and reliable VCTE measurements exhibited VCTE-F0/F1 at M3.

Congruently, CAP as a parameter of liver steatosis significantly decreased 3 months after MBS. While non-invasive staging of liver steatosis using CAP is difficult, particularly in (still) obese subjects, and different cutoffs have been proposed in patients with MASLD^29–31^, in our cohort there was a significant decrease in non-invasively assessed liver steatosis stage, no matter which system of CAP cutoffs was applied in our cohort. Of note, there was also a significant decrease in LSM and CAP at M3 when only analyzing patients with MASH, underscoring the potential benefit of MBS particularly in these patients by early improvement of MASH already early after MBS. These findings are in line with previous studies showing regression of MASLD/MASH in patients with obesity after MBS^10,44^.

The importance of MBS in the treatment of MASLD/MASH in patients with obesity is increasing in light of escalating incidence^1,2^. Recently published findings of a multicenter randomized trial suggest the superiority of MBS compared to lifestyle intervention and optimal medical care after a 1-year observational period^13^. Additionally, evidence indicates that the resolution of MASLD/MASH following MBS is enduring, as demonstrated by other authors who reported substantial percentages of 84% of MASLD/MASH resolution after a 5-year period^10^. Our findings supplement the existing knowledge regarding the speed at which these changes manifest. In this brief 3-month observational period, significant improvements in MASLD/MASH were already evident. This signifies that MBS may be capable of rapidly improving hepatic steatosis/inflammation.

Furthermore, importantly, this study provides evidence that development of liver dysfunction or even liver cirrhosis after MBS is uncommon and that, on the contrary, liver chemistries including ALT and GGT improved further even within the normal range. Since many patients with MASLD/MASH exhibit transaminases within the normal range^45^, this decrease of ALT might indicate a rapprochement to a “true normal range” in these patients.

On the other hand, there was a slight increase of bilirubin in patients after bariatric procedures. Moreover, there was one patient developing hepatocellular and cholestatic liver injury 3 months after MBS, respectively. While our data does not suggest that liver injury or liver dysfunction is particularly prevalent after MBS, liver chemistries should be controlled regularly in all patients undergoing metabolic/bariatric procedures, as liver dysfunction and chronic liver injury has been reported in some individuals^15^.

This study has some limitations. Firstly, due to organizational reasons during the SARS-CoV-2 (COVID-19) pandemic^46^, not every parameter was available for all patients. This was especially relevant for LSM at follow-up. Apart from the missing values, this reduced the sample size for these parameters, which represents the major limitation of our study. Secondly, it is important to note that this study was conducted as an observational study. The selection of the surgical method was based on factors such as BMI, patient preference, comorbidities (excluding liver health), and the presence of reflux. The consideration of MASLD/MASH status did not play a role in determining the choice between surgical methods. Moreover, this longitudinal study investigated the non-invasive parameters of liver stiffness and liver steatosis over a course of three months after MBS. As such, we did not directly investigate changes in liver fibrosis and liver fat content and cannot derive from our data whether the short-term changes in non-invasive parameters of liver fibrosis and steatosis truly reflect a regression of fibrosis and steatosis in MASLD or reflect the dynamics of other confounding factors such as inflammation, liver congestion, blood flow or cholestasis. Notably, while MASLD and MASH were prevalent in the cohort included in this study, no patient exhibited advanced fibrosis or liver cirrhosis. Moreover, our study only examined short-term changes after MBS. Further studies are required to analyze the trajectory of liver stiffness, liver steatosis and liver chemistries after MBS over a longer period of time. Finally, we did not examine the associations between liver histology features and non-invasive parameters of liver fibrosis and liver stiffness, as this was already done by a previous study^47^.

In conclusion, in this prospective observational study, we thoroughly investigated the short-term trajectory of various non-invasive markers of liver stiffness and liver steatosis in a cohort of obese patients undergoing MBS with a high prevalence of MASLD and MASH. Importantly, LSM—as assessed by both VCTE and SWE—and fibrosis stage decreased significantly 3 months after MBS. Furthermore, liver steatosis as assessed by CAP declined with not only a significant decrease in median CAP, but also a regression of steatosis stage. Importantly, comparable findings were obtained in the subgroup of patients with MASH. This denotes that MBS may be a viable treatment for obese patients with MASLD/MASH patients facilitating rapid improvement of hepatic steatosis and inflammation, although further research is required to confirm our findings. Finally, assessing the trajectory of liver chemistries, we showed that liver injury does occur, but is infrequent after MBS procedures. Importantly, no patient in our study developed hepatic dysfunction after MBS. Still, regular postoperative laboratory check-up of liver chemistries is warranted to detect liver injury early and be able to enact countermeasures in a timely fashion.

### Supplementary Information

Supplementary Information.

## Data Availability

The data is available upon reasonable request to the corresponding author.

## Abbreviations

2DSWE: 2D shear wave elastography
ALT: Alanine aminotransferase
AST: Aspartate aminotransferase
APRI: AST to platelet ratio index
MBS: Metabolic/bariatric surgery
BL: Baseline
BMI: Body mass index
CAP: Controlled attenuation parameter
CDC: Clavien Dindo classification
CLD: Chronic liver disease
cACLD: Compensated advanced chronic liver disease
COVID19: SARS-CoV-2
EWL%: Excess weight loss (in percentage)
TWL%: Total weight loss (in percentage)
GGT: Gamma glutamyl transferase
HbA1c: Glycated hemoglobin (in percentage)
IQR: Interquartile range/median
LSM: Liver stiffness measurement
M: Medium
M3: 3 Months
MASLD: Metabolic dysfunction-associated steatotic liver disease
MASH: Metabolic dysfunction-associated steatohepatitis
NAS: Non-alcoholic fatty liver disease activity score
N: Number
NIT: Non-invasive tests
OAGB: One anastomosis gastric bypass
RYGB: Roux-en-Y gastric bypass
SADI-S: Single anastomosis duodeno-ileal bypass with sleeve gastrectomy
SG: Sleeve gastrectomy
SWE: Shear wave elastography
pSWE: Point shear wave elastography
ULN: Upper limit of normal
VCTE: Vibration-controlled transient elastography
XL: Extra-large
