# Supplementary material for: Rapid improvement of hepatic steatosis and liver stiffness after metabolic/bariatric surgery: a prospective study
Source: Sci Rep. 2024 Jul 30;14:17558. doi: 10.1038/s41598-024-67415-w (PMC11289378; doi:10.1038/s41598-024-67415-w)
Supplement: Supplementary file 1 — Supplementary Information. [file 41598_2024_67415_MOESM1_ESM.docx]

# SUPPLEMENTARY MATERIAL

RAPID IMPROVEMENT OF HEPaTIC STEATOSIS AND liver stiffness after METABOLIC/BARIATRIC SURGERY – a Prospective study

**Larissa Nixdorf_1_#**

**Lukas Hartl_2_#**

Stefanie Ströhl_2_

Daniel Moritz Felsenreich_1_

Magdalena Mairinger_1_

Julia Jedamzik_1_

Paula Richwien_1_,

Behrang Mozayani_3_

Georg Semmler_2_

Lorenz Balcar_2_

Michael Schwarz_2_

Mathias Jachs_2_

Nina Dominik_2_

Christoph Bichler_1_

Michael Trauner_2_

Mattias Mandorfer_2_

Thomas Reiberger_2_

Felix B Langer_1_

**David Josef Maria Bauer_2_***

**Gerhard Prager_1_***

_1_ Division of Visceral Surgery, Department of General Surgery, Medical University of Vienna, Vienna, Austria

_2_ Division of Gastroenterology and Hepatology, Department of Medicine III, Medical University of Vienna, Vienna, Austria

_3_ Department of Pathology, Medical University of Vienna, Vienna, Austria.

# authors share first authorship

* authors share last authorship

Correspondence: Prof. Dr. Thomas REIBERGER

Division of Gastroenterology and Hepatology

Department of Medicine III

Waehringer Guertel 18-20, A-1090 Vienna, Austria

P: +4314040065890 F: +4314040047350

M: thomas.reiberger@meduniwien.ac.at

**TABLE OF CONTENTS**

FIGURES

Figure-S1. Comparison of liver stiffness measured by (A) VCTE, (B) pSWE and (C) 2D SWE, as well as (D) CAP, MASLD fibrosis index and alanine aminotransferase (ALT) at baseline and after 3 months in patients with MASH.

Page 3

TABLES

Table-S1. Cutoffs of controlled attenuation parameter (CAP) for the diagnosis of the stage of liver steatosis before metabolic/bariatric surgery (MBS) among patients with reliable CAP at baseline (n=80).

Page 4

Table-S2. Short-term course of laboratory parameters after bariatric surgery

Page 5

Table-S3. Trajectory of LSM, CAP and laboratory parameters after metabolic/bariatric surgery in patients with metabolic dysfunction-associated steatohepatitis (MASH).

Page 7

Table-S4. Prevalence of liver injury at baseline and 3 months after metabolic/bariatric surgery.

Page 9

REFERENCES

Page 10

**FIGURES**

**Figure-S1. Comparison of liver stiffness measured by (A) VCTE, (B) pSWE and (C) 2D SWE, as well as (D) CAP, MASLD fibrosis index and alanine aminotransferase (ALT) at baseline and after 3 months in patients with MASH.**

*Abbreviations: 2DSWE = 2D share wave elastography; BMI = body mass index; CAP = controlled attenuation parameter; LSM = liver stiffness measurement; pSWE = point share wave elastography;*


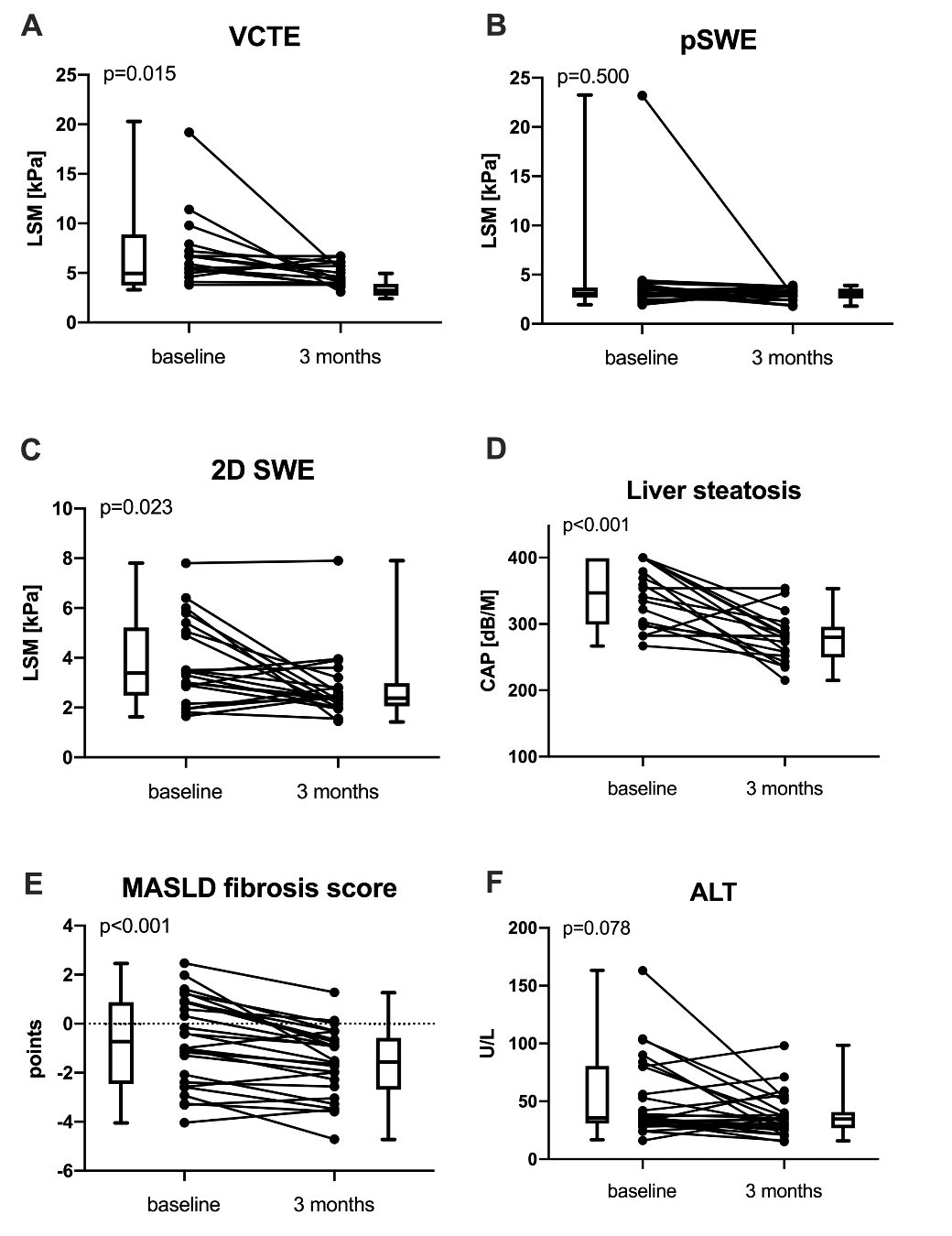


**Table-S1. Cutoffs of controlled attenuation parameter (CAP) for the diagnosis of the stage of liver steatosis before metabolic/bariatric surgery (MBS) among patients with reliable CAP at baseline (n=80).** Correlations were assessed using Spearman’s Rho.

| **Patient characteristics** | **Non-invasive steatosis stage** | **n (%)** | **Correlation with histological steatosis** | **p-value** |
| --- | --- | --- | --- | --- |
| **Karlas et al. (1)** | 0 | 9 (11.3%) | 0.312 | **0.005** |
|  | 1 | 5 (6.2%) |  |  |
|  | 2 | 3 (3.8%) |  |  |
|  | 3 | 63 (78.7%) |  |  |
| **Runge et al. (2)** | 0 | 11 (13.8%) | 0.265 | **0.017** |
|  | 1 | 12 (15.0%) |  |  |
|  | 2 | 18 (22.5%) |  |  |
|  | 3 | 39 (48.7%) |  |  |
| **Naveau et al. (3)** | 0 | 29 (36.3%) | 0.221 | **0.049** |
|  | 1 | 12 (15.0%) |  |  |
|  | 2 | 6 (7.5%) |  |  |
|  | 3 | 33 (41.3%) |  |  |

**Table-S2. Short-term course of laboratory parameters after bariatric surgery**

*Abbreviations: ALT = alanine aminotransferase; APRI = AST to platelet ratio index; AST = aspartate aminotransferase; GGT = gamma glutamyl transferase; HbA1c = glycated hemoglobin; INR = international normalized ratio; MASLD = metabolic dysfunction-associated liver disease;*

| **Patient characteristics** | **at baseline** | **after 3 months** | **p-value** |
| --- | --- | --- | --- |
| **AST, U x L^-1^ (IQR)^1^** | 24.0 (19.3-33.0) | 23.0 (19.0-31.0) | 0.785 |
| **ALT, U x L^-1^ (IQR)^2^** | 34.0 (24.8-51.5) | 31.0 (23.8-39.3) | **0.025** |
| **GGT, U x L^-1^ (IQR)^3^** | 30.0 (24.0-43.3) | 21.0 (15.8-33.0) | **<0.001** |
|  |  |  |  |
| **APRI score, points (IQR)^4^** | 0.2 (0.2-0.3) | 0.2 (0.2-0.3) | 0.974 |
| **MASLD fibrosis score, points (IQR)^4^** | -0.97 (-2.08-0.71) | -1.74 (-2.60- -0.78) | **<0.001** |
|  |  |  |  |
| **Bilirubin, mg x dL^-1^ (IQR)^5^** | 0.5 (0.4-0.6) | 0.6 (0.4-0.8) | **<0.001** |
| **Albumin, g x L^-1^ (IQR)^5^** | 40.2 (37.3-41.9) | 42.3 (40.2-44.8) | **<0.001** |
| **Platelet count, G x L^-1^ (IQR)^5^** | 258.0 (224.0-305.5) | 253.0 (222.0-283.0) | 0.211 |
| **INR, units (IQR)^6^** | 1.1 (1.0-1.1) | 1.1 (1.0-1.2) | **0.048** |
| **Sodium, mmol x L^-1^ (IQR)^5^** | 139.0 (138.0-141.5) | 142.0 (140.0-143.0) | **<0.001** |
| **Creatinine, mg x dL^-1^ (IQR)^5^** | 0.7 (0.6-0.9) | 0.7 (0.6-0.8) | **0.002** |
|  |  |  |  |
| **Fasting glucose, mg x dL^-1^ (IQR)^7^** | 86.5 (77.5-109.8) | 88.0 (84.0-99.5) | 0.844 |
| **HbA1c, % (IQR)^8^** | 5.6 (5.2-6.7) | 5.3 (5.0-5.7) | **<0.001** |
| **Cholesterol, mg x dL^-1^ (IQR)^1^** | 143.0 (126.0-168.0) | 142.0 (115.0-160.5) | 0.107 |

^1^ in patients with available longitudinal values (n=77)

^2^ in patients with available longitudinal values (n=78)

^3^ in patients with available longitudinal values (n=79)

^4^ in patients with available longitudinal values (n=76)

^5^ in patients with available longitudinal values (n=81)

^6^ in patients with available longitudinal values (n=75)

^7^ in patients with available longitudinal values (n=80)

^8^ in patients with available longitudinal values (n=47)

**Table-S3. Trajectory of LSM, CAP and laboratory parameters after metabolic/bariatric surgery in patients with metabolic dysfunction-associated steatohepatitis (MASH).**

*Abbreviations: ALT = alanine aminotransferase; APRI = AST to platelet ratio index; AST = aspartate aminotransferase; GGT = gamma glutamyl transferase; HbA1c = glycated hemoglobin; INR = international normalized ratio; CAP = controlled attenuation parameter; LSM = liver stiffness measurement; MASLD = metabolic dysfunction-associated liver disease;*

| **Patients with MASH** | **at baseline** | **after 3 months** | **p-value** |
| --- | --- | --- | --- |
| **LSM by vibration-controlled transient elastography, kPa (IQR)^1^** | 6.3 (5.1-7.7) | 4.5 (3.9-6.0) | **0.015** |
| **LSM by point shear wave elastography, kPa (IQR)^2^** | 3.1 (2.7-3.7) | 3.0 (2.6-3.6) | 0.500 |
| **LSM by 2D shear wave elastography, kPa (IQR)^2^** | 3.4 (2.5-5.2) | 2.4 (2.1-3.0) | **0.023** |
| **Controlled attenuation parameter, dB x m^-1^ (IQR)^3^** | 347.5 (299.5-400.0) | 280.0 (250.0-296.5) | **<0.001** |
|  |  |  |  |
| **APRI score, points (IQR)^4^** | 0.3 (0.2-0.4) | 0.2 (0.2-0.3) | 0.588 |
| **MASLD fibrosis score, points (IQR) ^4^** | -0.7 (-2.4 – 0.9) | -1.5 (-2.7 - -0.6) | **<0.001** |
|  |  |  |  |
| **AST, U x L^-1^ (IQR)^4^** | 24.5 (21.0-37.0) | 23.5 (20.0-34.0) | 0.495 |
| **ALT, U x L^-1^ (IQR)^5^** | 35.0 (30.0-80.0) | 34.0 (26.0-40.0) | 0.078 |
| **GGT, U x L^-1^ (IQR)^5^** | 33.0 (26.0-51.0) | 24.0 (18.0-33.0) | **<0.001** |
| **Bilirubin, mg x dL^-1^ (IQR)^6^** | 0.5 (0.4-0.6) | 0.6 (0.5-0.8) | **<0.001** |
| **Albumin, g x L^-1^ (IQR)^6^** | 39.7 (38.4-44.6) | 42.8 (41.3-44.5) | **0.009** |
| **Platelet count, G x L^-1^ (IQR)^6^** | 260.5 (214.5-336.5) | 261.0 (233.5-298.5) | 0.674 |
| **INR, units (IQR)^5^** | 1.1 (1.0-1.1) | 1.1 (1.1-1.2) | **0.016** |
| **Creatinine, mg x dL^-1^ (IQR)^4^** | 0.8 (0.7-0.9) | 0.7 (0.6-0.8) | **0.022** |
|  |  |  |  |
| **Fasting glucose, mg x dL^-1^ (IQR)^6^** | 98.0 (81.5-123.0) | 95.0 (86.0-101.0) | 0.153 |
| **HbA1c, % (IQR)^7^** | 5.6 (5.2-6.7) | 5.3 (5.0-5.7) | **<0.001** |
| **Cholesterol, mg x dL^-1^ (IQR)^5^** | 137.0 (120.0-159.0) | 126.0 (110.0-156.0) | 0.298 |

^1^ indicated as median of reliable longitudinal VCTE measurements. Available in n=16 patients

^2^ indicated as median of longitudinal pSWE/2DSWE measurements. Available in n=21 patients

^3^ indicated as median of reliable longitudinal CAP measurements. Available in n=18 patients

^4^ in patients with available longitudinal values (n=26)

^5^ in patients with available longitudinal values (n=27)

^6^ in patients with available longitudinal values (n=28)

^7^ in patients with available longitudinal values (n=17)

**Table-S4. Prevalence of liver injury at baseline and 3 months after metabolic/bariatric surgery.**

*Abbreviations: ALT = alanine aminotransferase; AST = aspartate aminotransferase; GGT = gamma glutamyl transferase; ULN = upper limit of normal*

| **Parameter** | **at baseline** | **after 3 months** | **p-value** |
| --- | --- | --- | --- |
| **AST >3xULN, yes (%)^1^** | 0 (0.0%) | 1 (1.3%) | 0.999 |
| **ALT >3xULN, yes (%)^2^** | 1 (1.3%) | 2 (2.6%) | 0.999 |
| **Hepatocellular liver injury, yes (%)^2^** | 1 (1.3%) | 2 (2.6%) | 0.999 |
|  |  |  |  |
| **GGT >2xULN, yes (%)^3^** | 2 (2.5%) | 2 (2.5%) | 0.999 |
| **Bilirubin >2xULN, yes (%)^4^** | 0 (0.0%) | 1 (1.2%) | 0.999 |
| **Cholestatic liver injury, yes (%)^4^** | 2 (2.5%) | 3 (3.7%) | 0.999 |

^1^ longitudinally available in n=77 patients

^2^ longitudinally available in n=78 patients

^3^ longitudinally available in n=79 patients

^4^ longitudinally available in n=81 patients

**REFERENCES**

1. Karlas T, Petroff D, Sasso M, Fan JG, Mi YQ, de Lédinghen V, Kumar M, et al. Individual patient data meta-analysis of controlled attenuation parameter (CAP) technology for assessing steatosis. J Hepatol 2017;66:1022-1030.

2. Runge JH, Smits LP, Verheij J, Depla A, Kuiken SD, Baak BC, Nederveen AJ, et al. MR Spectroscopy-derived Proton Density Fat Fraction Is Superior to Controlled Attenuation Parameter for Detecting and Grading Hepatic Steatosis. Radiology 2018;286:547-556.

3. Naveau S, Voican CS, Lebrun A, Gaillard M, Lamouri K, Njiké-Nakseu M, Courie R, et al. Controlled attenuation parameter for diagnosing steatosis in bariatric surgery candidates with suspected nonalcoholic fatty liver disease. Eur J Gastroenterol Hepatol 2017;29:1022-1030.
